# Antidiabetic Effect of Methanolic Extract from *Berberis julianae* Schneid. via Activation of AMP-Activated Protein Kinase in Type 2 Diabetic Mice

**DOI:** 10.1155/2014/106206

**Published:** 2014-09-02

**Authors:** Jing Yang, Ping Zhao, Dingrong Wan, Qi Zhou, Chao Wang, Guangwen Shu, Zhinan Mei, Xinzhou Yang

**Affiliations:** ^1^College of Pharmacy, South-Central University for Nationalities, 182 Min-Zu Road, Wuhan 430074, China; ^2^College of Life Sciences, South-Central University for Nationalities, 182 Min-Zu Road, Wuhan 430074, China

## Abstract

We have investigated the antidiabetic effect and mechanism of methanolic extract of *Berberis julianae* Schneid. (BJSME) in STZ induced Type 2 diabetes mellitus mice. T2DM mice were induced by high fat diet and low dose streptozotocin (STZ). BJSME was orally administrated at the doses of 60, 120, and 240 mg/kg/d, for 21 days. Metformin was used as positive control drug. Food intake, body weight, plasma glucose, oral glucose tolerance test, insulin tolerance test, insulin, and blood-lipid content were measured. The effects of BJSME on the glucose transporter 4 (GLUT4) translocation in L6 myotubes and the GLUT4 protein expression in skeletal muscle as well as phosphorylation of the AMP-activated protein kinase (AMPK) in liver and muscle were examined. *In vitro* and *in vivo* results indicate that BJSME increased GLUT4 translocation by 1.8-fold and BJSME significantly improved the oral glucose tolerance and low density lipoprotein cholesterol (LDL-C) of serum and reduced body weight, glucose, and other related blood-lipid contents. The BJSME treatment also stimulated the phosphorylation of AMPK. Thus, BJSME seems to possess promising beneficial effects for the treatment of T2DM with the possible mechanism via stimulating AMPK activity.

## 1. Introduction

Diabetes mellitus (DM) is a metabolic disorder of the endocrine system. DM may lead to various complications such as renal failure, cardiovascular disease, blindness, or non-fatty liver disease [1]. The number of people diagnosed with diabetes increased so dramatically all over the world in the last twenty years. According to World Health Organization (WHO) projections, the diabetic population is likely to increase to 300 million or more by the year 2025 [2]. Type 2 diabetes mellitus (T2DM) comprises 90% of people with diabetes around the world [3, 4], and insulin resistance is the typical feature of T2DM [5]. Currently available therapies for T2DM include insulin and various oral antidiabetic agents such as sulfonylureas, biguanides, *α*-glucosidase inhibitors, and glinides, which are used as monotherapy or in combination to achieve better glycemic regulation [6, 7]. Many of these oral antidiabetic agents have a number of serious adverse effects [7]. Managing diabetes without any side effects is still a challenge. Therefore, the search for more effective and safer natural hypoglycemic agents has continued to be an important area of investigation. The traditional Chinese medicines (TCMs) have been used to counteract DM (recognized as “Xiao Ke Zheng” in ancient China) for thousand years based on unique theory system with few side effects, and it has been attracting more and more attention for its dialectical therapy [8].

The glucose transporter 4 (GLUT4), as one of the 13 sugar transporter proteins (GLUT1–GLUT12, and HMIT), is highly expressed in adipose tissue and skeletal muscle and catalyzes hexose transport across cell membranes [9]. It is a major mediator of glucose removal from the circulation and a key regulator of whole-body glucose homeostasis. GLUT4 translocation promoting glucose uptake is vital to glucose homeostasis and is a defined target of antidiabetic drug research [10, 11].

In this study, a cell-based GLUT4 translocation system developed in L6 myotubes coexpressing recombinant GLUT4 and IRAP using confocal imaging technique was established to screen the extracts or fractions from TCMs for discovery of novel antidiabetic agents to fight T2DM. During the screening of a TCM extract library (400 biotas) on GLUT4 translocation in myotubes, we found a methanolic extract of the traditional Chinese medicine,* Berberis julianae* Schneid., which has long been used for the treatment of infection and hypertension [12], displayed promising positive activity on GLUT4 translocation.

In the present study, it was shown that the methanolic extract of* Berberis julianae* Schneid. (BJSME) significantly improved the oral glucose tolerance and insulin tolerance, increased the number of pancreatic islets, and reduced fat and liver mass, adipose accumulation in liver, blood glucose, and related blood-lipid content without altering body weight and food intake in T2DM mice. BJSME increased GLUT4 expression in skeletal muscle and GLUT4 translocation in L6 myotubes. The BJSME treatment also stimulated the activity of the AMP-activated protein kinase (AMPK) in liver and skeletal muscle.

## 2. Methods and Materials

### 2.1. Preparation of the Methanolic Extract from* Berberis julianae* Schneid. 

The fresh roots of* B. julianae* Schneid. were collected in August 2013 at Longping, Jianshi county, Hubei province, China, and identified by Professor Dingrong Wan of College of Pharmacy, South-Central University for Nationalities. A plant voucher specimen (number 20130825BJ) was deposited in the Herbarium of South-Central University for Nationalities, Wuhan, China. Air-dried roots of* B. julianae* Schneid. (500 g) were ground and then extracted sequentially by maceration at room temperature with methanol (3 × 2.5 L, 3 h each). The solvents were evaporated at reduced pressure to yield 35.7 g of the residue.

### 2.2. Cell Culture

L6 cells were cultured in minimum essential medium alpha modification (MEM-*α*, Hyclone, USA) supplemented with 10% fetal bovine serum (FBS, Hyclone, USA) and 1% antibiotics (100 U/mL penicillin and 100 *μ*g/mL streptomycin) at 37°C in 5% CO_2_. For differentiation into myotubes, cells were cultured in MEM-*α* supported with 2% FBS at 37°C in 5% CO_2_ and the medium was replaced every 48 h. L6 cells were used for experiment 7 days after differentiation.

### 2.3. IRAP Translocation Assay

L6 cells were transected with pIRAP-mOrange cDNAs (presented by Professor Xu Tao, Chinese Academy of Sciences) using Lipofectamine 2000 as per manufacturers protocol. L6 cells stably expressing IRAP-mOrange (L6 IRAP-mOrange) were cultured in *α*-MEM supplemented with 10% fetal bovine serum and 1% antibiotics (100 U/mL penicillin and 100 *μ*g/mL streptomycin) at 37°C in 5% CO_2_.

Before starting the experiment, L6 IRAP-mOrange was seeded in forty-eight-well plates, and incubated until 100% confluence and then starved in serum-free *α*-MEM for 2 h. The cells were imaged with a laser-scanning confocal microscope LSM 510 (Carl Zeiss, Jena, Germany) to monitor the dynamics of IRAP-mOrange translocation. Images were taken after addition of 100 nM insulin or 10 *μ*g/mL test samples, using 555 nm excitation laser every 10 seconds in first 2 minutes and then every 5 minutes in later 25 min.

### 2.4. Animals and Treatments

Male KM mice (*n* = 80) weighing between 18 and 22 g were approved by the Hubei Provincial Center for Disease Control and Prevention [certification number SCXK (E) 2008-0005]. The animals were housed at 22 ± 2°C, 45–75% relative humidity, where 12 h dark-light cycles were maintained with free access to food and water. Before the experiments, the mice had been acclimatized to the laboratory conditions for one week. All the animal experimental procedures were performed in accordance with International Guidelines for Care and Use of Laboratory Animals and approved by the Animal Ethical Committee of the Institute of Health and Epidemic Prevention (Wuhan, China).

Mice were then randomly assigned to receive either the standard chow diet as the normal control group (*n* = 10) or a high fat diet (containing 35% carbohydrate, 20% protein, and 45% fat, Medicience Ltd., Yangzhou, China) (*n* = 70) for 4 weeks. Then, the mice fed with high fat diet were injected with streptozotocin (STZ, Sigma-Aldrich, St. Louis, USA) [120 mg/kg, intraperitoneal injection (i.p.)]. Seven days later, the fasted blood glucose levels of the mice were tested. Fifty mice whose fasted blood glucose levels ≥11.1 mmol/L were classified as T2DM [13]. These mice were fed with high fat diet in later study.

Normal control group received vehicle. Fifty T2DM mice were randomly divided into five groups (*n* = 10): one of the groups was treated with vehicle; the second was treated with metformin (200 mg/kg/d, China Associated Pharmaceutical Co., Ltd., Shenzhen, China); the rest three groups were treated with BJSME at the dose of 60 mg/kg/d, 120 mg/kg/d, or 240 mg/kg/d, respectively. Metformin and BJSME were dissolved in 0.9% normal saline. The mice were treated with the solution orally daily with a gastric tube for 21 days.

### 2.5. Body Weight, Food Intake, and Fasted Blood Glucose Levels

The body weight and food intake were daily recorded. Fasted blood glucose levels were measured weekly by a blood glucose meter (ONETOUCH, Ultra, Lifecan, USA) [14].

### 2.6. Oral Glucose Tolerance Test and Insulin Tolerance Test

An insulin tolerance test was performed in all of the animals after 18 days of treatment. Glucose levels were tested at 0, 30, 60, and 120 min by a blood glucose meter (ONETOUCH, Ultra, Lifecan, USA) after a 2 IU/kg insulin intraperitoneal injection [14].

An oral glucose tolerance test was performed in mice after 12 h fasting at the 20th day of treatment. Blood glucose taken from the tail tip at 0, 30, 60, and 120 min after glucose administration was measured using a blood glucose meter (ONETOUCH, Ultra, Lifecan, USA). The glucose load was 2 g/kg orally [15].

### 2.7. Biochemical Analysis

At the end of the experiment, the mice were fasted for 12 h. Blood samples were collected by retroorbital sinus puncture using capillary tubes under diethyl ether anesthesia. Then, serums were prepared by centrifuging the blood samples at 3000 rpm for 15 min. The serum levels of insulin, triglycerides (TG), total cholesterol (TC), free fatty acids (FFA), low density lipoprotein cholesterol (LDL-C), and high density lipoprotein cholesterol (HDL-C) were determined by corresponding assay kits (Jiancheng Bioengineering Institute, Nanjing, China).

Then, the mice were euthanized; livers, skeletal muscles, and other tissues were harvested. After weighing, part of livers and pancreas were immediately stored in liquid nitrogen tank, and the rest were fixed in 10% neutral buffered formalin, embedded by paraffin, and then stained in hematoxylin and eosin. The stained tissues were observed through an optical microscope and were photographed.

The levels of TC, TG, and FFA in mice livers or skeletal muscle tissues were determined by the method described previously [16].

### 2.8. Western Blot Analysis

The livers were lysed in 1x RIPA buffer (50 mM Tris-HCl [p H8], 150 mM NaCl, 1% NP-40, 0.5% sodium deoxycholate, 1% SDS), supplemented with complete protease inhibitor cocktail (Roche) and phosphatase inhibitor (Phosstop, Roche), and then centrifuged at 10000 rmp, 4°C for 12 min. Supernatant protein concentration was determined with BCA assay kit (Abgent). Same amounts of protein were then diluted in SDS sample buffer (Tris-HCl, glycerol, SDS, DTT, and bromophenol blue), subjected to 10% SDS-PAGE, and immunoblotted with antibodies specific for AMPK*α*, phospho-AMPK-*α* (Thr172) (Cell Signaling Technology).

The skeletal muscles were lysed in 1x RIPA buffer (50 mM Tris-HCl [pH 8], 150 mM NaCl, 1% NP-40, 0.5% sodium deoxycholate, 1% SDS), supplemented with complete protease inhibitor cocktail (Roche), and then centrifuged at 2500 rpm for 10 min, 4°C; supernatant protein concentration was determined with BCA assay kit (Abgent). Same amounts of protein were then diluted in SDS sample buffer (Tris-HCl, glycerol, SDS, DTT, and bromophenol blue), loaded them onto 10% SDS-PAGE, and immunoblotted with antibodies specific for GLUT4 (Cell Signaling Technology). Before loading samples, incubate them at 65°C for 10 min.

### 2.9. Statistical Analysis

One-way ANOVA was used for multiple group comparisons. Correlation analysis was carried out using Pearson's correlation analysis. Data was shown as means ± standard error (*M* ± SEM). *P* values < 0.05 were considered significant. Statistical analyses were performed using the GraphPad Prism 5.0 software package.

## 3. Results

### 3.1. Effects of BJSME on IRAP Translocation Quantity in Cells

**Figure 1:**
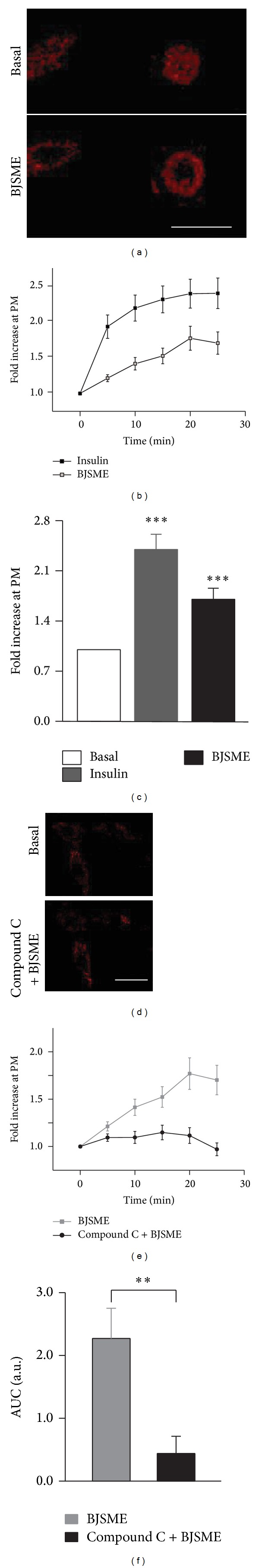
BJSME stimulated IRAP trafficking in L6 cell. (a) L6 cells were infected with pIRAP-mOrange in order to detect externalized IRAP by confocal microscopy. Confocal images in L6 cells incubated in the absence (basal) or presence of BJSME for 25 minutes. Scale bar: 40 *μ*m. (b) Time course of the change in fluorescence at PM induced by insulin (*n* = 25 cells) and BJSME (*n* = 22 cells) in IRAP-mOrange transfected L6 cells. Data represent three independent experiments. (c)* M* ± SEM of the change in fluorescence from (b). Data represent the fold increase in fluorescence induced by insulin and BJSME between 0 and 25 minutes. IRAP-mOrange surface labeling in experiments was measured in L6 cells under insulin and BJSME-treated conditions. Data represent* M* ± SEM of values from three separate experiments. ***P < 0.001 compared to basal. (d) Confocal images in L6 cells incubated in the absence (basal) or presence of Compound C and BJSME for 25 minutes. Scale bar: 40 *μ*m. (e) Time course of the change in fluorescence induced by BJSME (*n* = 22 cells) and Compound C with BJSME (*n* = 19 cells) in IRAP-mOrange transfected L6 cells. Data represent three independent experiments. (f) Mean ± SEM of arbitrary units of the change in fluorescence from (e). Data represent the fold increase in fluorescence induced by Compound C and BJSME from 0 to 25 minutes. The fluorescence of L6 cells treated by BJSME compared with BJSME in Compound C has significant difference (**P < 0.01).

In order to acquire the potential activity of BJSME on glucose metabolism, we first examined its effect on the translocation of the IRAP to the cell plasma membrane (PM). We used confocal microscopy to specifically examine the trafficking of enhanced red fluorescent protein- (mOrange-) tagged IRAP in L6 cells. As shown in Figure 1, these cells were highly BJSME responsive in terms of BJSME-regulated IRAP translocation. The addition of BJSME had obvious effect on the translocation of IRAP (Figure 1(a)). As we know, insulin can cause a time-dependent increase in GLUT4-EGFP fluorescence in the evanescent field. This is consistent with the time course for insulin-stimulated IRAP translocation to the PM in L6 cell. We found that the BJSME also could stimulate IRAP translocation to PM in L6 cell. Meanwhile, we found that the addition of insulin or BJSME had significant effect on the kinetics (Figure 1(b)) or magnitude (Figure 1(c)) of the IRAP trafficking response. In our experiments, we assume BJSME-induced transportation of IRAP may be through AMPK pathway. In order to prove this, we incubated L6 cells with adding of Compound C (an inhibitor of AMPK) for 30 minutes and then added BJSME. We found BJSME could not increase the translocation of IRAP with the prior adding of Compound C by confocal microscopy (Figure 1(d)), compared with BJSME-direct stimulation. It showed that Compound C can completely inhibit the AMPK pathway and prevent the IRAP translocation caused by BJSME (Figures 1(e) and 1(f)).

### 3.2. Effects of BJSME on Body Weight, Food Intake and Tissue Weight, Fasted Blood Glucose, Serum Insulin Levels, OGTT, and ITT

**Figure 2:**
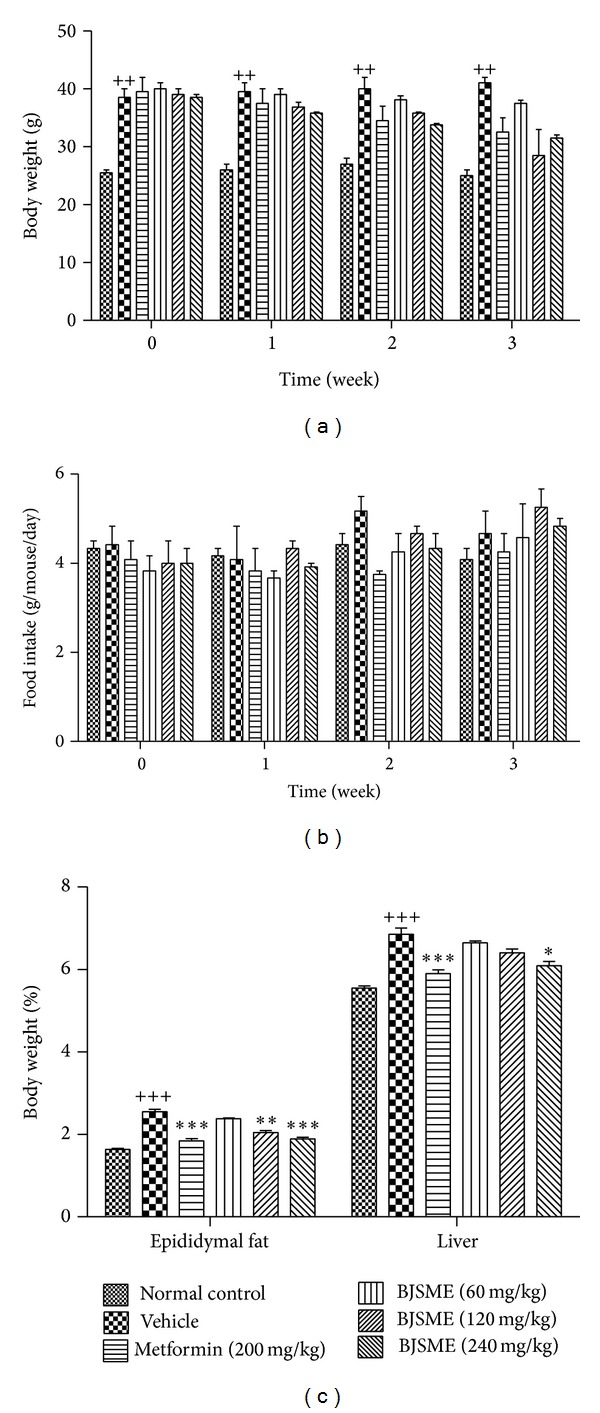
Body weight (a) and food intake (b) during the treatment. (c) At the end of the study, weight of epididymal fat and liver to total body weight. ^+++^ *P* ≤ 0.001, ^++^ *P* ≤ 0.01 compared to normal control; ***P ≤ 0.001, **P ≤ 0.01, and *P ≤ 0.05, compared to T2DM mice treated with vehicle.

As shown in Figures 2(a) and 2(b), there were no significant differences in body weight and food intake between vehicle groups and BJSME groups during the 21 days of BJSME treatment, while fat and liver mass indicated a clear reduction (Figure 2(c)).

**Figure 3:**
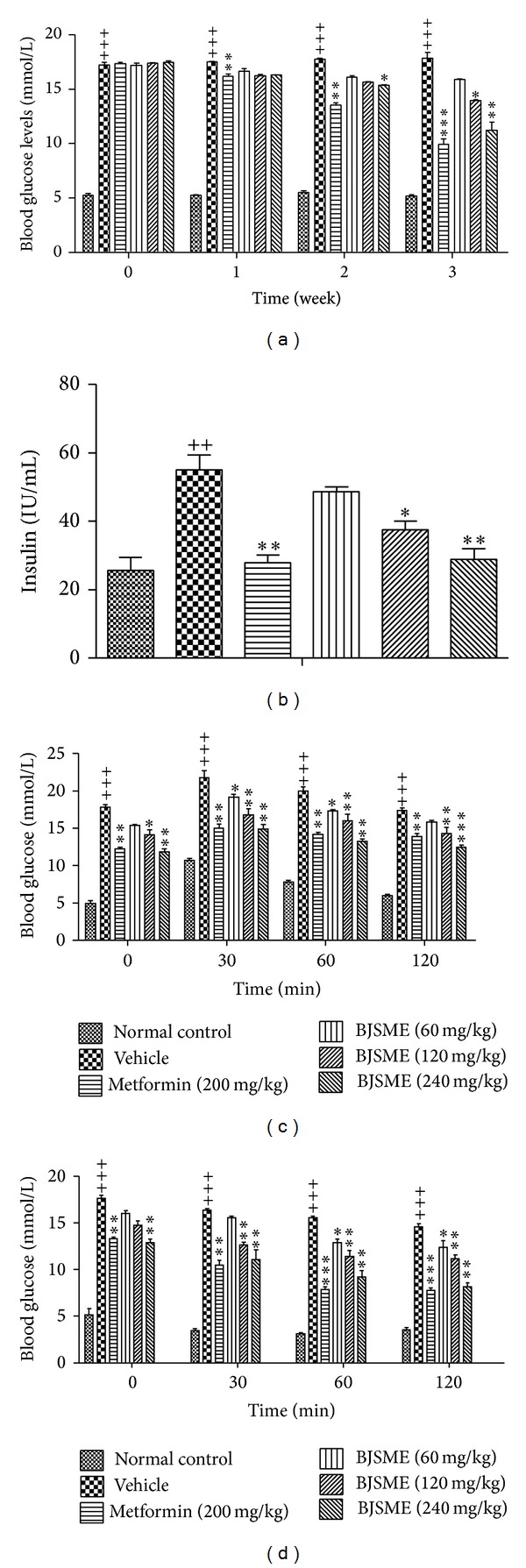
(a) Effect of BJSME on fasted blood glucose levels. (b) Effect of BJSME on serum insulin levels at the end of the 21-day treatment. (c) Effect of BJSME on OGTT. (d) Effect of BJSME on ITT. ^+++^ *P* ≤ 0.001, ^++^ *P* ≤ 0.01 compared to normal control; **P ≤ 0.01, *P ≤ 0.05, compared to T2DM mice treated with vehicle.

Plasma glucose concentration, measured after an overnight fast, has been the mainstay of diagnosing diabetes for a century [17]. However, it has been debated that the cutoff values should be considered diagnostic. The history of oral glucose tolerance test (OGTT) is instructive in this regard. In our study, BJSME groups resulted in a significant reduction in fasted blood glucose levels (Figure 3(a)) and serum insulin levels (Figure 3(b)) compared with an obvious improvement in OGTT (Figure 3(c)), and the effects were dose-dependent. To assess insulin sensitivity in T2DM mice treated with BJSME, we performed insulin tolerance test (ITT). The ITT (Figure 3(d)) had been improved.

### 3.3. Effects of BJSME on Serum Lipid Profile and Tissue Lipid Content

**Figure 4:**
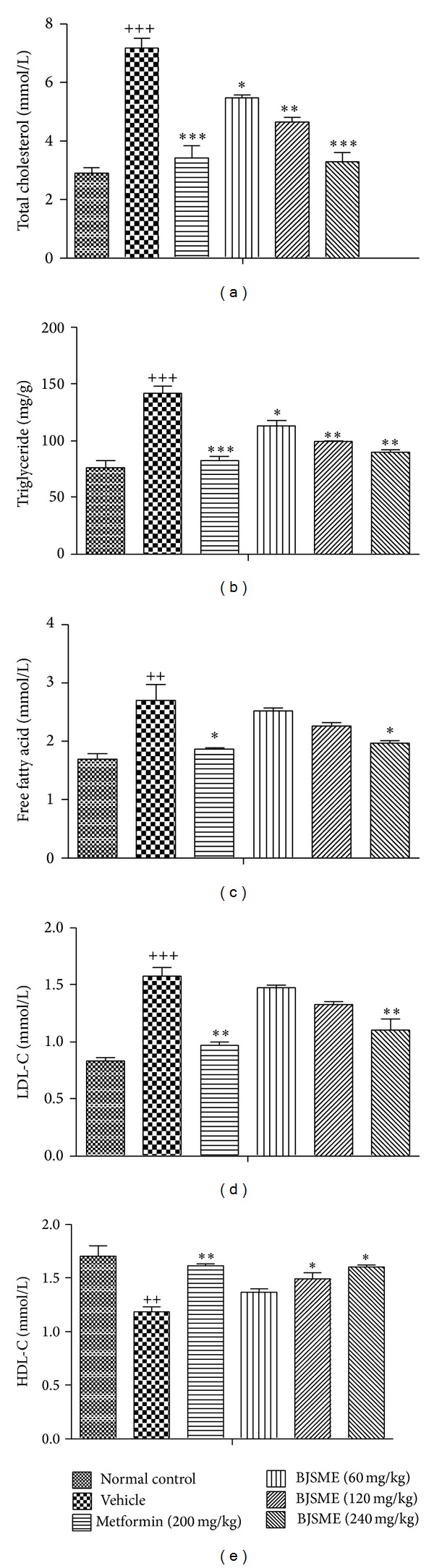
Effects of BJSME on TC (a), TG (b), FFA (c), LDL-C (d), and HDL-C (e) levels in rat serum at the end of the study; ^+++^ *P* ≤ 0.001, ^++^ *P* ≤ 0.01 compared to normal control; ***P ≤ 0.001, **P ≤ 0.01, and *P ≤ 0.05 compared to T2DM mice treated with vehicle.

**Figure 5:**
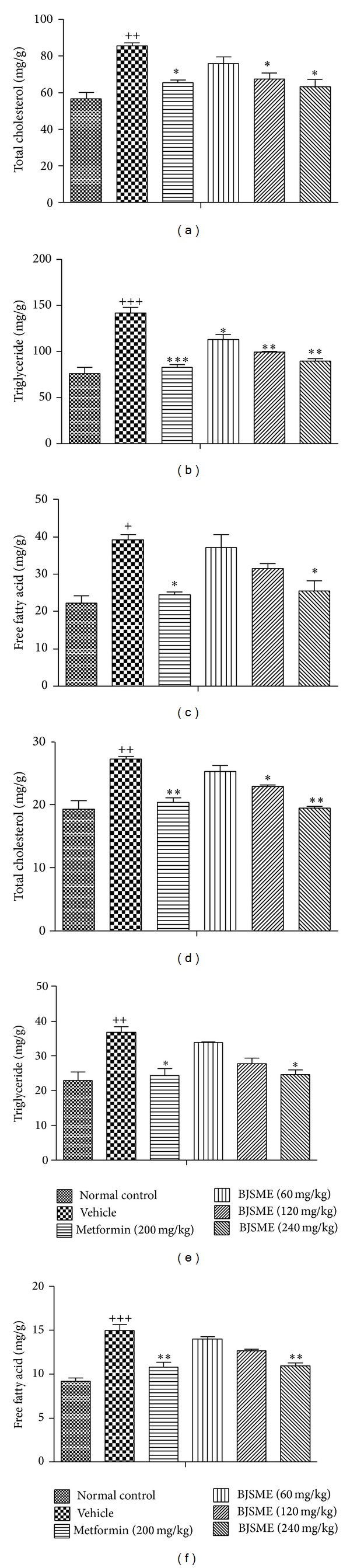
Effects of BJSME on TC (a), TG (b), and FFA (c) levels in mice liver tissue. Effects of BJSME on TC (d), TG (e), and FFA (f) levels in mice skeletal muscle tissue. ^+++^ *P* ≤ 0.001, ^++^ *P* ≤ 0.01, and ^+^ *P* ≤ 0.05, compared to normal control; **P ≤ 0.01, *P ≤ 0.05 compared to T2DM mice treated with vehicle.

**Figure 6:**
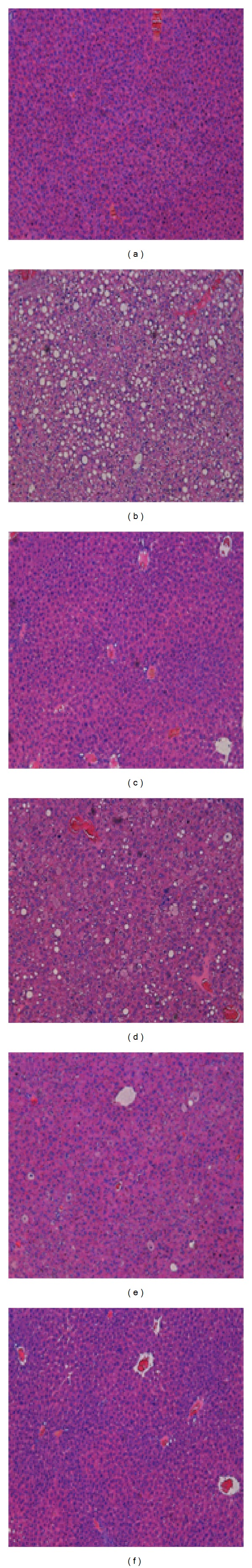
Histological sections of mouse livers. Optic microscopy: HE (200x). (a) Normal control. (b) T2DM mice treated with vehicle. (c) T2DM mice treated with metformin (200 mg/kg). (d) T2DM treated with BJSME (60 mg/kg). (e) T2DM treated with BJSME (120 mg/kg). (f) T2DM treated with BJSME (240 mg/kg).

Hyperlipidemia and tissue steatosis are two risk factors leading to cardiovascular disease [18]. Thus, we examined the effects of BJSMF on serum lipid parameters and adipose accumulation in liver and skeletal muscle in all mice.  Figure 4 showed the effects of BJSME on serum lipid content. The results showed that BJSME significantly reduced the levels of serum TC, TG, FFA, and LDL-C and increased the serum HDL-C level. After 21-day treatment, the content in tissue also significantly decreased (Figure 5). Adipose accumulation in the livers could be observed clearly in the vehicle group T2DM mice, which was reduced in the T2DM mice treated with BJSME (Figure 6).

### 3.4. Effects of BJSME on Histopathology of Pancreas

**Figure 7:**
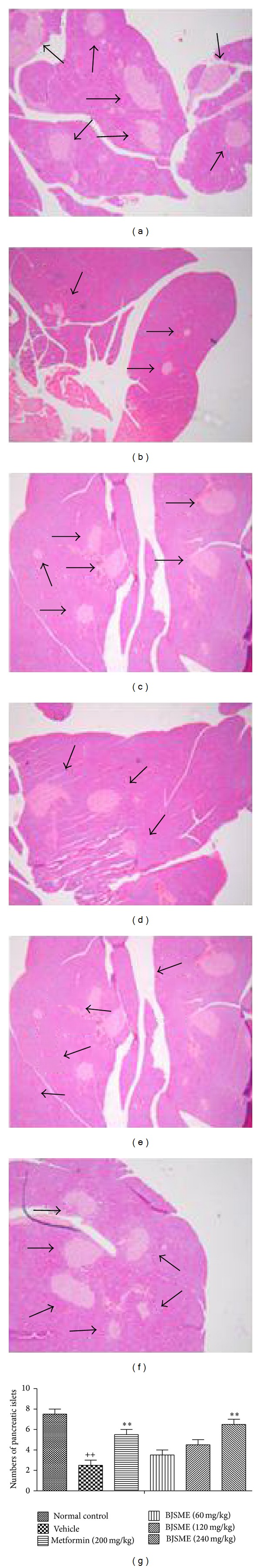
Effects of BJSME on morphological features of mice pancreas. Optic microscopy: HE (100xs). (a) Normal control. (b) T2DM mice treated with vehicle. (c) T2DM mice treated with metformin (200 mg/kg). (d) T2DM mice treated with BJSME (60 mg/kg). (e) T2DM mice treated with BJSME (120 mg/kg). (f) T2DM mice treated with BJSME (240 mg/kg). (g) The statistical results of the number of pancreatic islets per microscope field. ^++^ *P* ≤ 0.01, compared to normal control; **P ≤ 0.01, compared to T2DM mice treated with vehicle.

In our study, low-dose STZ-induced moderate pancreatic *β*-cell damage is a significant event initiating hyperglycemia. To determine whether BJSME was able to defend pancreatic *β*-cells, we performed histological examinations with HE staining. The results showed that STZ treatment obviously decreased the number of pancreatic islets in T2DM mice and this change was relieved by BJSME treatment (Figure 7).

### 3.5. Western Blot Analysis

**Figure 8:**
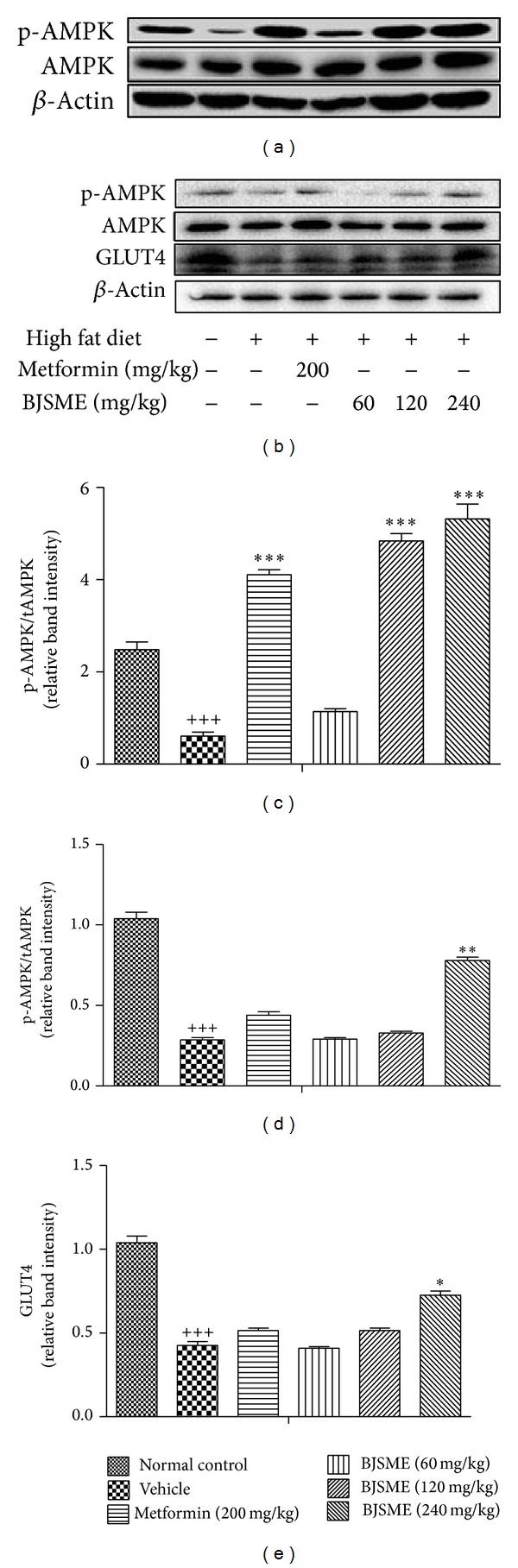
Effect of BJSME on AMPK phosphorylation in liver (a). Effects of BJSME on AMPK phosphorylation and GLUT4 in skeletal muscle (b). (c)–(e): quantitative comparisons of expression levels of p-AMPK/AMPK in liver, p-AMPK/AMPK in skeletal muscle, and GLUT4 in skeletal muscle. ^+++^ *P* ≤ 0.001, compared to normal control; ***P ≤ 0.001, **P ≤ 0.05, and *P ≤ 0.01 compared to T2DM mice treated with vehicle.

AMPK is activated under a variety of conditions that signify cellular stress, usually in response to a change in the intracellular ATP-to-AMP ratio. Active AMPK orchestrates a variety of metabolic processes, most of which lead to reduced energy storage and increased energy production [19]. To determine whether the effects of BJSME in animals could activate AMPK, we examined the phosphorylation of AMPK in liver. As shown in Figures 8(a) and 8(b), it was obvious that AMPK phosphorylation was increased in liver.

Both protein levels and insulin-stimulated translocation of GLUT4 are reduced in the muscle of diabetic human subjects. Due to the important role of GLUT4 in insulin action* in vivo* and GLUT4 dysfunction in the diabetic states, therapeutics that promote GLUT4 translocation could increase postprandial glucose uptake into skeletal muscle, consequently improving insulin sensitivity [20]. In our experiment, the expression of GLUT4 in skeletal muscle of T2DM mice had been improved (Figure 8(b)).

## 4. Discussion

GLUT4 is the most important glucose transporter in skeletal muscle glucose metabolism [21], and this transporter is highly expressed in cell types that exhibit regulated glucose uptake, such as adipocytes, skeletal muscle cells, and cardiomyocytes. GLUT4 plays a key role in whole-body glucose homeostasis. It is reported that mice deficient in GLUT4 developed Type 2 diabetes, whereas transgenic mice (overexpressing GLUT4) glucose metabolism has been improved* in vivo* [22–24]. In the diabetic db/db mouse model, overexpression of the human GLUT4 gene protected these animals from insulin resistance and diabetes [25]. Interestingly, the global GLUT4−/− homozygous knockout mice showed a less severe phenotype with respect to glucose homeostasis than heterozygous GLUT4+/− mice, most likely due to compensatory mechanisms in the homozygous condition [26]. In addition, the GLUT4−/− mice showed many severe defects, including decreased life spans and growth retardation, as well as cardiac and adipose tissue abnormalities and further complicating interpretations [25]. The heterozygous GLUT4+/− mice, however, suffered from insulin resistant and were predisposed to develop into diabetes [27]. In addition, when the GLUT4 gene was specifically ablated in muscle tissue, insulin resistance and glucose intolerance were observed in mice as young as 8 weeks [23]. Furthermore, ablation of GLUT4 specifically in adipose tissue resulted in impaired glucose uptake in both muscle and adipose tissues [28]. Interestingly, the liver also developed insulin resistance, which suggested that the GLUT4-deficient adipose tissue secreted one or more factors that travel via the bloodstream to liver and muscle tissues. Thus, although the global disruption of GLUT4 yielded a complicated mouse phenotype with multiple defects, it is clear from the tissue-specific knockouts that the presence of GLUT4 specifically in muscle and adipose tissue is critical for maintaining normal whole-body glucose homeostasis [29]. The above considerations could place GLUT4 as a novel therapeutic target for the treatment of Type 2 diabetes in human.

It has been shown that the subcellular distribution of GLUT4 plays an important role in glucose homeostasis in muscle and adipocyte. Three main proteins stored in GSV are GLUT4, IRAP, and Sortilin. In adipocyte which has differentiated for three days, IRAP transfers from the donor membrane into GSV; however, in undifferentiated cells, the limited expression Sortilin cannot recruit IRAP to GSV [30]. But the overexpression Sortilin and GLUT4 can reconstitute the functional GSV which fused with the original IRAP. It is reasoned that, in the process of cell differentiation, the Sortilin recruits GLUT4 to the GSV, and IRAP is recruited by GLUT4 [31].

Some studies have proved that IRAP gene knockout mice (IRAP−/− mice) are with normal insulin levels, but the function of response to insulin is impaired. The total amount of GLUT4 expression in all tissues (such as muscle, heart, and fat) was also reduced to 40%~85% [32]. When IRAP gene silencing in adipocyte, the increased exocytosis leads to the enhancing of GLUT4 on the membrane, indicating that the IRAP plays a fundamental role in GLUT4 cycle [33]. Besides, another major finding was that the IRAP mutation mice would cause heart enlargement, which is similar to mice whose GLUT4 expression were absent or decreased [34]. This explains that IRAP shows colocalization relationship with GLUT4. Thus, detecting the IRAP can indirectly reflect the situation of GLUT4. Therefore, developing a cell-based GLUT4 translocation system in L6 myotubes coexpressing recombinant GLUT4 and IRAP could be a stable and efficient screening method for the discovery of novel antidiabetic agents to fight Type 2 diabetes.

In this study, we have established a screening method to preliminarily identify plant extracts, fractions, and their isolated compounds with potential antidiabetic activity through assessing their effect on the translocation of GLUT4 to PM. During a screening program, we found that BJSME exhibited a strong effect to stimulate GLUT4 translocation by 1.8-fold in L6 cells, as insulin displayed 2.3-fold. Based on the finding* in vitro*, we predicted that BJSME may have the antidiabetic potency* in vivo*. Our* in vivo* data clearly showed the antidiabetic activity of BJSME, including the amelioration of hyperglycemia and hyperinsulinemia. We observed that BJSME significantly alleviated hyperglycemia and hyperinsulinemia in T2DM mice. OGTT and ITT further indicated that hyperglycemia and hyperinsulinemia were considerably ameliorated by BJSME. T2DM patients are often prone to suffering from cardiovascular diseases as a result of dyslipidemia [35]. IR, a hallmark of T2DM, is potential to promote the development of lipid accumulation in hepatocyte through impairing the capacity of insulin to repress lipolysis. This event leads to elevated circulating FFA and lipid accumulation in livers [36, 37]. In addition, skeletal muscle is another important insulin-responsive tissue besides liver. A very strong connection between IR in T2DM and skeletal muscle steatosis has been reported [38]. In our present study, we observed a significant elevation of TG, TC, and FFA levels in serums, livers, and skeletal muscles of T2DM mice. In another aspect, level of HDL-C in serum was downregulated in T2DM animals. These abnormalities could be alleviated by BJSME treatment via a dose-dependent manner. Histopathological examinations of mouse livers supplied the evidence that BJSME dose-dependently and efficiently rescued liver steatosis associated with T2DM. Moreover, the hypoglycemic activity of BJSME was similar to metformin.

AMP-activated protein kinase (AMPK) is an evolutionarily conserved guardian of cellular and systemic energy metabolism. As a cellular energy regulator, AMPK plays a major role in glucose and lipid metabolism and in the control of metabolic disorders such as diabetes, obesity, and cancer [39, 40], and it has emerged as a therapeutic target for metabolic disorders [41]. Activation of AMPK increases fatty acid oxidation, inhibits lipid synthesis, and can improve insulin action [42, 43]. Based on a number of studies showing that AMPK regulates a variety of different metabolic pathways, it is widely recognized as a useful and safe target for the treatment of metabolic disorders such as T2DM and dyslipidemia [44]. Furthermore, AMPK pathway is a major regulatory pathway of GLUT4 translocation which is an essential step for inducible glucose uptake into muscle and fat. In our study, we observed that the AMPK activity was increased by BJSME, suggesting BJSME with therapeutic potential for diabetes by targeting AMPK.

## 5. Conclusions

In this study, we developed a cell-based GLUT4 translocation system for the discovery of novel antidiabetic agents against Type 2 diabetes in L6 myotubes coexpressing recombinant GLUT4 and IRAP using confocal imaging technique. Based on the screening method, the methanolic extract of the traditional Chinese ethnic medicine,* Berberis julianae* Schneid., displayed promising positive activity on GLUT4 translocation. The further* in vitro* and* in vivo* study showed that BJSME improved insulin sensitivity, reduced hyperglycemia, and resumed insulin levels, at least in part, by activating GLUT4 translocation. Moreover, the GLUT4 translocation may be modulated by AMPK. Our work suggested that the cell-based GLUT4 translocation system in L6 myotubes coexpressing recombinant GLUT4 and IRAP could be an efficient and effective screening method for the discovery of new antidiabetic agents from nature resources, and BJSME deserves further investigation for possible antidiabetic drug development.
